# Confocal micro X-ray fluorescence analysis for the non-destructive investigation of structured and inhomogeneous samples

**DOI:** 10.1007/s00216-023-04829-x

**Published:** 2023-07-24

**Authors:** Korbinian Heimler, Christine Gottschalk, Carla Vogt

**Affiliations:** 1 Institute of Analytical Chemistry, TU Bergakademie Freiberg, Leipziger Str. 29, 09599 Freiberg, Germany; 2 Present Address: AMINO GmbH, An der Zucker-Raffinerie 9, 38373 Frellstedt, Germany

**Keywords:** Confocal micro X-ray fluorescence analysis, Confocal, XRF, Three-dimensional, Depth profile, Non-destructive

## Abstract

Confocal micro X-ray fluorescence (CMXRF) spectroscopy is a non-destructive, depth-resolved, and element-specific technique that is used to analyze the elemental composition of a sample. For this, a focused beam of mono- or polychromatic X-rays is applied to excite the atoms in the sample, causing them to emit fluorescence radiation which is detected with focusing capillary optics. The confocal design of the instrument allows for depth-resolved analysis, in most cases with a resolution in the lower micrometer dimension after collecting X-rays from a predefined volume within the sample. The element-specific nature of the technique allows information to be obtained about the presence and concentration of specific elements in this volume. This makes CMXRF spectroscopy a valuable tool for a wide range of applications, especially when samples with an inhomogeneous distribution of elements and a relatively light matrix have to be analyzed, which are typical examples in materials science, geology, and biology. The technique is also commonly used in the art and archaeology fields to analyze the elemental composition of historical artifacts and works of art, helping to provide valuable insights into their provenance, composition, and making. Recent technical developments to increase sensitivity and efforts to improve quantification in three-dimensional samples will encourage wider use of this method across a multitude of fields of application in the near future.

**Graphical abstract:**

Confocal micro X-ray fluorescence (CMXRF) is based on the confocal overlap of two polycapillary lens foci, creating a depth-sensitive and non-destructive probing volume. Three-dimensional resolved element distribution images can be obtained by measuring the fluorescence intensity as function of the three-dimensional position.

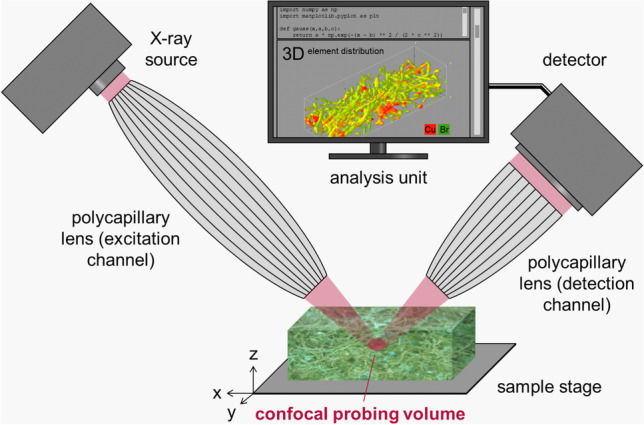

## Introduction

In various fields of application, a growing interest and demand for three-dimensional characterization of the elemental composition of structured materials can be observed, but only a limited selection of analytical techniques and methods fulfills these rising requirements with high spatial resolution. In this context, versatile analysis methods, modifications, and enhancements of the device designs were applied during the past years in order to extend 2D methods to make them capable of performing depth-sensitive analysis. These devices differ not only in their technical functionality, but also in the range of information generated, quantification approaches, and device-related measurement parameters such as sensitivity or resolution. In this respect, also a very fundamental differentiation can be drawn between destructive and non-destructive analysis techniques. Destructive methods are based on the progressive removal of the sample volume during the analysis, as is the case for secondary ion mass spectrometry (SIMS) [1, 2], laser ablation inductively coupled plasma mass spectrometry (LA-ICP-MS) [3, 4], or laser-induced breakdown spectroscopy (LIBS) [4, 5]. Alternatively, a repeated surface preparation or slicing and subsequent analysis of the samples is possible, in which case the subsequently applied analytical method also could be non-destructive [6, 7]. Fully non-destructive methods are mostly based on the use of high-energy radiation (keV) or particles (MeV) that have the advantage of not only multiple measurements and follow-up analyses but also application to samples that should remain intact, such as art-historically significant objects (archaeometry). Some examples of non-destructive or quasi-nondestructive and feasible three-dimensionally resolving methods are computed tomography (CT) [3, 6–9], prompt gamma neutron activation analysis (PGAA) [10], particle-induced X-ray emission (PIXE) [11, 12], X-ray absorption fine structure (XAFS) [12–15], and micro X-ray fluorescence (MXRF) spectroscopy. Most of these techniques work with spot sizes on the middle or lower micrometer scale, but could be distinguished by the different ways which are applied to generate the analytical signal (photons, ions, neutrons) and the characteristics of the resulting spectra (analyzable elements, resolution, background, sensitivity). Therefore, each of the analytical techniques has its own selection of samples for which the technique will be ideally suited.

This paper focuses on confocal micro X-ray fluorescence (CMXRF) spectroscopy as a non-destructive and depth-sensitive analysis method. In the past 20 years, significant developments in CMXRF spectroscopy have been contributed by various research groups around the world. CMXRF is a type of spectrometer that uses X-ray fluorescence to determine the composition of a sample at a high spatial resolution at the microscale. This makes it particularly useful for studying small samples or for analyzing samples with complex or inhomogeneous compositions. The confocal aspect of the technique refers to the use of a confocal aperture to focus the X-ray beam, allowing for high spatial resolution and the ability to measure depth-dependent information within the sample. This makes it possible to obtain detailed information about the distribution of elements within the sample and to study the structural and compositional changes that may occur within.

CMXRF is commonly applied in a variety of fields, including materials science, archaeometry, biology, and geology. In materials science, it can be used to study the elemental composition of metals and alloys, as well as ceramics and polymers. The results are used to better understand the properties and behavior of these materials and to facilitate the development of new and improved materials for a variety of applications. In archaeometry, CMXRF can be used to analyze artifacts and other archaeological samples in order to gain insight into the provenance of the materials and objects, and the technologies used in their making, thus helping the archaeologists to better understand the history and cultural significance of these objects. In biological samples, the technique can be applied to study the distribution and concentration of elements within living organisms. This can provide valuable information about the function of different tissues and organs, as well as the effects of various diseases and treatments. In geology, CMXRF can be used to study the elemental composition of rocks and minerals to provide insight into the geological processes that formed these materials, as well as their potential use as natural resources.

In the following, the operating principle, technically relevant aspects, the advantages of this technique, and the challenges for the 3D analysis of structured materials will be illustrated by a variety of results from current fields of application, starting with the fundamental components.

## X-ray fluorescence spectroscopy

The discovery of X-rays in 1895 by W. C. Röntgen [16] and the transillumination of the human body marked the beginning of the use of X-rays, and with the findings of Laue in 1912 [17] on X-ray diffraction (XRD) and Moseley in 1913 [18] on the relationship between atomic number and wavelength, the foundations were laid for the use of X-ray spectroscopy. XRF spectroscopy now is both a qualitative and a quantitative method for non-destructive material analysis and enables the measurement of powders and solid compact samples as well as liquids. The method is employed routinely in the fields of mineralogy, metallurgy, glass, ceramics, and building materials industry or in coal and ore extraction. Particularly noteworthy is the application in archaeology, where it is important due to its capability for non-destructive analysis [19, 20]. In the typical energy range of 1–40 keV used by XRF, most elements are represented due to their emitted characteristic radiation (K and L lines) [19]. Exceptions have to be made for the light elements of the periodic table with Z < 11, since here the competing Auger effect leads to rather low fluorescence yields and low sensitivities in X-ray fluorescence measurements [20, 21]. Depending on the sample matrix, elements, and device design, concentrations in the lower ppm range (mg/kg) can be measured. The analytical method is suitable for the upper trace range, but is usually used in the concentration range from 0.01 to 100% [22].

## X-ray sources, fluorescence detection, and spectra evaluation

There are several types of X-ray sources that are commonly used in XRF analysis on the microscale, including X-ray tubes and synchrotron radiation sources. Each type of X-ray source has its own unique characteristics and advantages, and the choice of an X-ray source may depend on the specific requirements and constraints of the measurement.

Synchrotron radiation can be generated by synchrotron facilities, which are large and complex ring accelerators that use high-frequency electric alternating fields and powerful magnets to accelerate charged particles to near the speed of light on a circular path. This high-energy and high-intensity radiation is produced when the particles move through magnetic fields, which cause them to emit X-rays at specific wavelengths. Synchrotron radiation sources provide photon fluxes several orders of magnitude higher than X-ray tubes, and thus can provide higher sensitivity and resolution for XRF measurements [6, 15, 23–25]. However, they are much larger and more complex than X-ray tubes and require specialized facilities and expertise to operate.

Otherwise, the excitation radiation can be generated by the most common type of X-ray source used in XRF spectroscopy. X-ray tubes are relatively compact and can be easily integrated into laboratory spectrometers. However, they are limited in terms of the X-ray energy and intensity that can be generated (up to 50 W), which can affect the sensitivity and resolution of the measurements. These sources produce spectra with primary X-ray emission characteristic of the target (Cr, Mo, Rh, Ag, Au, or W is often used as anode material) as well as bremsstrahlung due to the deceleration process of charged particles. Especially in the context of MXRF analysis, so-called micro-focus X-ray tubes are generally used, as these sources already provide a quite useful focus of the X-ray beam [20, 22, 26–29].

The subsequent detection of the emitted radiation of the sample is often carried out by means of energy-dispersive spectrum processing (EDXRF). During the past years, silicon drift detectors (SDD) are mostly used for this, which are made of high-purity monocrystalline silicon wafers with a thickness of a few hundred microns [20, 30, 31]. It should be noted that so-called detector artifacts (interfering peaks) can overlay the spectrum and make the evaluation more difficult. These interference peaks include both sum and escape peaks, which result from X-ray photons arriving almost simultaneously or the excitation of the detector material by X-rays [32].

With the obtained XRF spectrum, qualitative and quantitative statements can be made, utilizing the position of the peak and the peak area. This evaluation can be performed with reference materials or the fundamental parameter method. A precise reference-material-based quantification requires matrix matching and on the microscale highly homogenous reference materials, which are in most cases not easily provided. Reference-free quantifications, on the other hand, are frequently based on the fundamental parameter method (FP method), which combines the complete mathematical description of the sample composition, the natural constants (fundamental parameters), and the instrument-specific parameters (based on the Sherman equation), and provide a mathematical relationship between the concentration and the measured photon number [33]. The following Sherman equation in case of a polychromatic excitation radiation (with the energy of the absorption edge ${\text{E}}_{\text{i}}^{\text{edge}}$ and the maximum energy of the X-ray tube ${\text{E}}_{\text{max}}$) correlates the mass fraction ${\text{c}}_{\text{Z}}$ of an element ${\text{Z}}$ in the sample with the fluorescence photon count ${\text{N}}_{\text{Z,i,j}}$ of fluorescence line $\text{i,j}$ of the element [30, 33]:

$${N}_{Z,i,j}={c}_{Z}{\int\nolimits}_{{E}_{i}^{{edge}}}^{{E}_{max}}\frac{{N}_{0}\left(E\right)}{{\overline{\mu }}_{Z,i,j}\left(E\right)}{\sigma }_{PP}^{Z,i-j}\left(E\right)dE\frac{{\Omega }_{\mathrm{det}}}{4\;\pi\; \mathrm{sin}\;\Psi }$$

The equation includes the influence of various parameters, like the excitation photon count ${\text{N}}_{0}\left({\text{E}}\right)$, the photo production cross section ${\sigma }_{PP}^{\text{Z,i-j}}\left({\text{E}}\right)$ (including the photo absorption cross section, absorption edge ratio, fluorescence yield, and transition probability), the effective mass attenuation coefficient ${\overline{\mu }}_{\text{Z,i,j}}\left({\text{E}}\right)$ (summarizing the absorption of excitation and fluorescence radiation), the angle of the excitation radiation to the sample surface $\psi$, and the solid angle of the detector $\frac{{\Omega }_{\mathrm{d}et}}{4\pi }$.

In the case of light matrices, such as in biological samples, polymers, and other organic-based materials, not all elements (such as H, C, or N) can be determined by means of XRF. Furthermore, scattering effects and higher-order effects (for example, when a released electron ionizes a neighboring atom) are not incorporated in the Sherman equation. The fundamental parameters and algorithms of the method are accordingly universal and not adapted to a specific sample. For the above reasons, the FP method is considered rather semi-quantitative, as it is less accurate than the reference-material-based quantification [19, 34].

Regarding XRF spectra evaluation in general, also scattering effects like the inelastic Compton scattering [35] and the elastic Rayleigh scattering [36] have to be considered. The latter leads to intensity losses due to the interaction between X-ray radiation and electrons and the formation of additional lines in the spectrum. Compared to the Rayleigh-scattered fluorescence lines of the sample, the intensity of the Compton-scattering lines in the spectrum increases with decreasing average *Z* of the matrix. Likewise, the continuous bremsstrahlung, which represents the main part of the spectral background, is scattered and also increases with a light sample matrix, resulting in a reduced peak-to-background ratio and reduced sensitivity [19, 30].

## Collimating X-ray optics and micro X-ray fluorescence spectroscopy

**Fig. 1:**
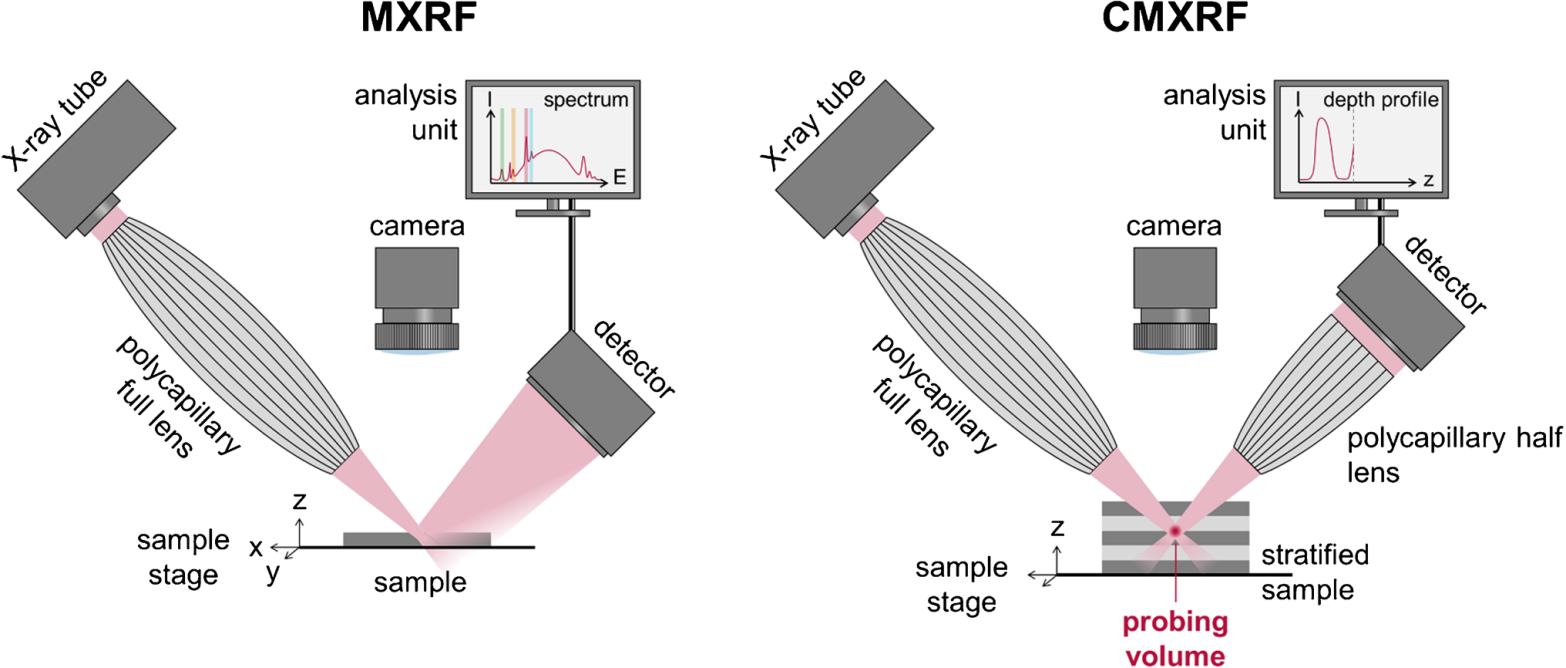
Schematic build-up of a MXRF and a CMXRF spectrometer (tabletop unit) for spatially resolved analysis. Focusing of X-rays by a polycapillary full lens down to a spot for surface analysis (left). Confocal overlapping of the foci of two X-ray optics creating a depth-sensitive probing volume (right) and sample movement by x-y-z motorized sample stage. Adapted from reference [27] with permission from the Royal Society of Chemistry

For focusing of X-rays, a large variety of technical solutions are applied at synchrotron facilities, like mono- and polycapillary X-ray optics, compound refractive lenses (CRLs), Kirkpatrick-Baez (KB) mirrors, Fresnel zone plates, and many more. In particular, the capillary X-ray optics feature a high versatility, since they can be applied for various X-ray sources [12]. The relatively low demands concerning installation and operation make these capillary X-ray optics a preferential tool for focusing X-rays in micro X-ray fluorescence spectrometers (MXRF) (Fig. 1).

MXRF is a matured analytical technique used to identify and measure the elemental composition of samples at the microscale, where a focused beam of X-rays is applied to excite the sample and lateral-resolved analysis is performed, which allows researchers to measure the elemental composition of the sample at different lateral positions within the field of view and can be used to study complex sample structures and compositions. For MXRF instruments, polycapillary lenses are usually applied, which consist of curved, hollow quartz glass capillaries, with the outer capillaries more curved than the inner ones [31]. In the polycapillary full lens, the X-ray radiation hits the polycapillary from a large spatial angle, the radiation is transported by multiple total reflection and focused on a small area. The requirements for this are very smooth capillary surfaces, a high reflection coefficient, and an incident radiation that hits the wall of the capillary at an angle that is smaller than the critical angle of total reflection. At the critical angle, the radiation proceeds parallel to the surface [31, 37, 38]. The X-rays that strike at a steeper angle are absorbed in the quartz material of the capillaries, and those which hit at a lower angle are reflected forward at an angle identical to that of the incident photons. The value of the critical angle is small in the X-ray range with mrad dimension [39]. The energy *E*, the density *ρ*, and the critical angle *θ*_crit_ are approximately related as follows [40]:

$${\theta }_{\mathrm{crit}}\approx \frac{20 \cdot \sqrt{\rho }}{E}$$

The greater the energy of the X-ray radiation, the smaller is the critical angle. In this case, transmission is the ratio of incident to outgoing radiation. This is higher in the medium energy range and decreases below 4 keV and above 20 keV. The reason for this is also the transmission property of the polycapillary optics. For lower energies, the acceptance angle (entrance angle) *θ*_in_ is larger than that for higher energies. This means that steeper entrance angles are possible for lower energies. In addition, the lower energy is transported better in the outer, more curved capillaries, which is an advantage of the geometry of a polycapillary lens, but this is disadvantageous with regard to the resulting longer path length. Longer path lengths lead to stronger absorptions, which result in a loss of intensity. For microscale resolution, the aim is to focus the radiation coming out of the polycapillary optic on one point (spot) [30, 31]. The spot size *D*_spot_ is approximately related to the diameter of the polycapillary lens at the lower end *D*_out_ and the critical angle via the following equation [31]:

$${D}_{\mathrm{spot}}={D}_{\mathrm{out}}+{2\theta }_{\mathrm{crit}}\cdot {d}_{2}$$

In general, smaller spot sizes lead to lower intensities on the sample and a small working distance *d*_2_ (distance lens to focus). With polycapillary lenses, a compromise between intensity, spot size, and working distance has to be achieved. A spot size of 15 to 25 μm results in an approximate working distance of 8 mm. This enables location-dependent analytics, making surface scanning methods such as line or area measurements (so-called line scans and mappings) possible in addition to point measurements. With mappings, element distribution images of surfaces of a sample can be acquired [34].

Depending on the orientation of a polycapillary half lens, either a focusing of parallel radiation or a parallel forwarding of bundled radiation can be achieved. In terms of basic principle and construction, it therefore corresponds to the full lens already described, in the start or end region of the lens [37, 40]. In CMXRF setups, a polycapillary half lens is often inserted as an additional X-ray optic between the sample and the detector to achieve the confocal geometry.

## Confocal micro X-ray fluorescence spectroscopy

The basic principles of the MXRF (Fig. 1, left) enable the lateral resolution of the elemental composition by surface, line, or point analysis. However, only limited statements can be made about elemental distributions in depth. In three-dimensional micro X-ray fluorescence analysis (3D-XRF; Fig. 1, right, currently referred to as confocal MXRF (CMXRF) [28]), this information is achieved by a confocal setup of X-ray optics in the excitation and detection channel and was implemented in the early 2000s [23, 24, 41–43]. Significant contributions have been made by various research groups such as Havrilla, Kanngießer, Streli, Sun, and Vincze, just to mention a few. Regarding CMXRF, a polycapillary lens is used in the excitation channel, complemented by an additional polycapillary half lens in the detection channel. The half lens allows the radiation to be collected from the focal point (better addressed as volume) and the parallelized radiation to be passed on to the detector. The field of view of the detector onto the sample is thus limited by the overlap of the foci of both polycapillary lenses (confocal design), which results in a microvolume from which the analytical information is obtained [24, 37, 40, 42]. The alignment process involves a careful adjustment of the positions and orientations of focusing optics in the excitation and detection channels to ensure that the focused beam of X-rays is accurately directed onto the sample and that the emitted fluorescence is collected and transmitted onto the detector. Afterwards, the depth-resolved and non-destructive analysis is realized by moving the sample through the microvolume of the fixed polycapillary lenses (Fig. 1). The depth of information of the described microvolume depends on the angle of incidence, angle of reflection, energy of the exciting radiation, energy of the fluorescence radiation, and the sample composition (matrix) [23]. This technique was initially mainly executed at synchrotron facilities (BAMline, BESSY II, beamline L of HASYLAB, and others) [23, 42, 43]. Here, the excitation takes place with monochromatic X-rays and polycapillary half lenses placed in the excitation and detection channel, respectively. Several synchrotron setups have been applied for the depth-resolved analysis of various samples until today [6, 13, 15, 25].

Further developments for X-ray optics and other device components allowed the application of the confocal setup also for tabletop systems with X-ray tubes instead of synchrotron radiation as excitation source. A confocal tabletop unit was already introduced in 2000 by Ding et al. for the analysis of radioactive materials for signal enhancement and background reduction [41]. In the following years, devices were developed for the realization of various depth-sensitive analysis methods [7, 29, 44–49]. As example, laboratory setups for measuring light elements by installing the spectrometer components inside a vacuum chamber and a monochromatic confocal tabletop setup were applied successfully at TU Vienna, Austria [49, 50]. Furthermore, spectrometers were also developed by various research groups from Belgium, China, Czech Republic, Germany, Japan, USA, and many more.

## Calibration and quantification of CMXRF spectrometers

Since the beginning of CMXRF spectroscopy in the early 2000s, multiple research groups worked on suitable quantification models, aiming for an advanced reconstruction of depth-resolved XRF data in 0D, 1D, 2D, and 3D dimensions. Fundamental to the development of any quantification at depth is the knowledge of various influencing parameters and effects. These include, for example, the distance to the sample surface, the composition of the sample in the excitation and detection beam paths, and the properties of the optics used. Since quantification is still a challenging task, in the following, common calibration methods applied in CMXRF spectrometry will be addressed by presenting recent quantitative routines for monochromatic and polychromatic excitation sources. Monochromatic measurements are mainly executed at synchrotron facilities by applying monochromator systems, whereas polychromatic radiation is produced by X-ray tubes for laboratory setups. Since energy transport within polycapillary optics is strongly energy dependent, different quantification routines need to be applied for both excitation types.

**Fig. 2:**
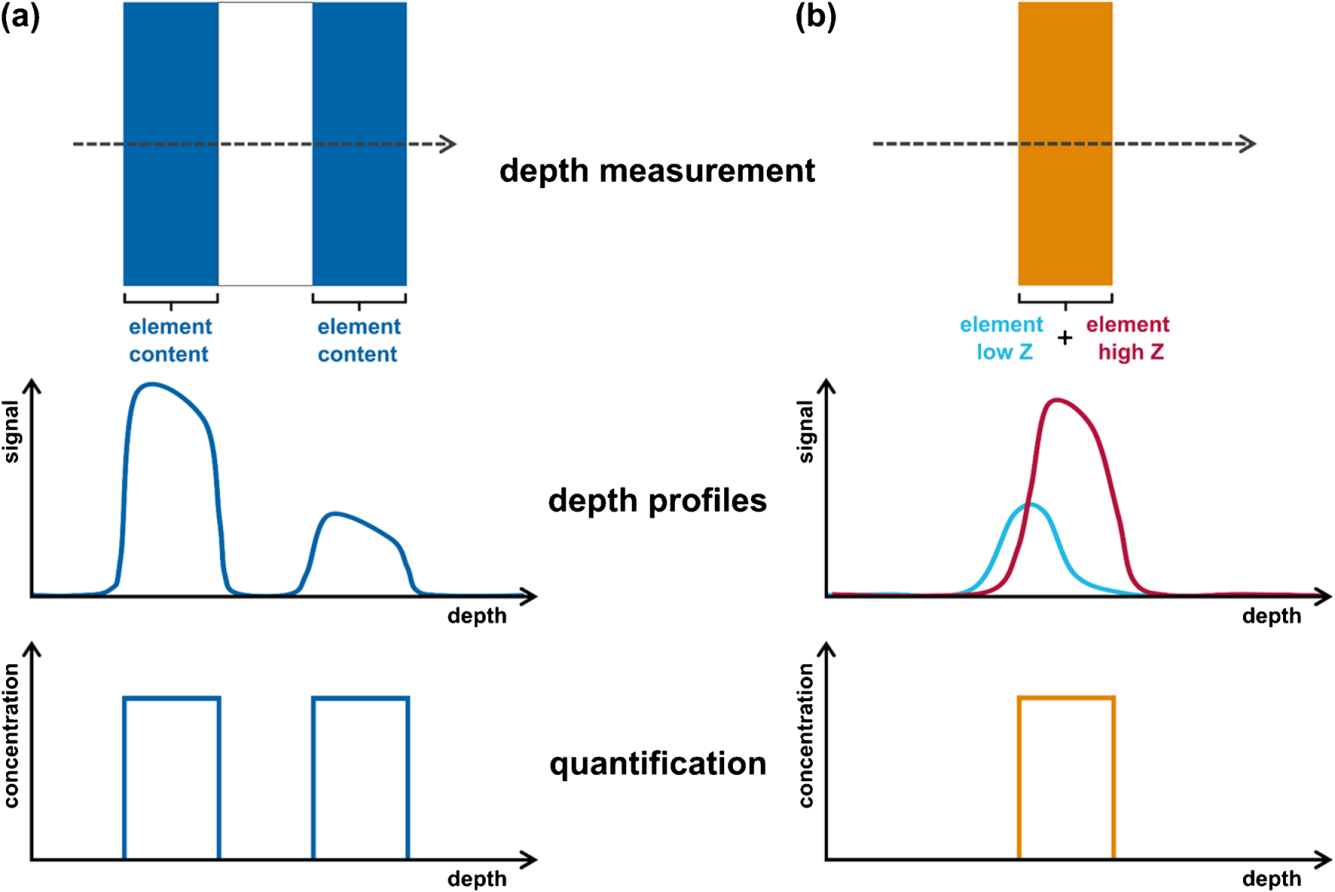
Schematic illustration of the challenges of quantification exemplified by layered samples. Hereby, the influence of, e.g., absorption effects (**a**) and atomic number *Z* (**b**) on the measured depth profiles is demonstrated. Those variables besides others need to be considered in the context of quantification of sample depth and element concentration

Already, the qualitative evaluation of CMXRF spectra is initially complicated by resolution and absorption effects that are dependent on the measured fluorescence energy of the X-ray peak. Two fluorescence energy effects should be mentioned in particular. The sample volume investigated in the focus of both capillaries decreases with increasing fluorescence energy. In the work of Mantouvalou et al. in 2010 [51], this was demonstrated on thin metal foils and, in 2012, on a glass reference standard with 200 µm thickness in which the elements Ca, Fe, Pb, and Sr were homogeneously distributed [52]. For this sample, the Sr Kα fluorescence line showed the smallest sample volume, as it has the highest fluorescence energy (Sr Kα 14.1 keV) of the four elements considered. This is caused by the effect of total reflection within the polycapillary lens in the detection channel, where, with increasing X-ray energy of the incident photons from the sample, the angle (or acceptance range) for total internal reflection decreases. As a result, radiation is collected from a smaller focal area (sample volume). The second effect relevant for spectral changes is the energy-dependent absorption by atoms in the sample. Photons coming from elements with low-energy fluorescence lines (e.g., Ca Kα 3.6 keV) are absorbed more strongly than those with higher energy. The depth of investigation is thus element- and matrix-dependent [51, 52]. Figure 2 illustrates those challenges in quantifying depth-dependent datasets in the context of simplified stratified samples. Sample (a) is a three-layer system with the same element concentration in the first and third layer (blue). The second layer is completely free from the element investigated. Sample (b) is a single-layer system (orange) containing two different elements of equal concentration. One element has a significantly lower atomic number (*Z*) (cyan) than the other one (red). In the first case (Fig. 2(a)), increasing absorption with depth results in steadily decreasing fluorescence signals. The steepness of the flanks depends on step width for the measurement and the sampling volume, which additionally depends on the atomic number of the investigated element. In the second case (Fig. 2(b)), depth profiles for both elements with the same concentration show effects of different sampling volume sizes and a different degree in absorption. Thus, the starting and ending points of both signals (cyan and red) measured in dependence of sample depth *z* do not appear at the same *z*-position and the intensity varies depending on the *Z* of the elements and resulting excitation and absorption effects.

Lin et al. developed a FP-based calculation for full 3D quantitative reconstruction for a synchrotron CMXRF setup, considering approaches described earlier by Szalóki et al*.* and others for the reconstruction of 2D elemental distribution concentrations [25, 53]. In this calculation, the actual spectrometer geometry and various emission paths were considered. By applying the Lambert-Beer law and describing the proportion factor of a certain X-ray fluorescence line with respect to the incident radiation as well as the attenuation factor and simplifying mathematical description, like neglecting secondary and higher-order effects, the calculation of the element concentration by the measured fluorescence intensity was achieved at any certain pixel in the 3D analysis space.

The depth resolution of the probing volume can be determined by measuring thin samples (thicknesses in the low micron range) of different elements, like several metal foils (e.g., Ti–Sr) [6, 15, 23, 25, 42]. It should be mentioned that this method of depth calibration is also quite commonly used for the laboratory CMXRF setups [54–56]. The energy-dependent depth resolution can be measured by plotting the X-ray intensities against the depth values. Since the probing volume inhabits a certain dimension, Gaussian-shaped depth profiles are detected (see schematic depth profiles in Fig. 2). The depth resolution can be calculated by the full width at half maximum (FWHM) value of the depth profiles FWHM_dp_ and the thickness of the sample *d*_foil_ which gives the full width at half maximum of the sampling volume FWHM_sv_ [25, 43].

$${\mathrm{FWHM}}_{\mathrm{sv}}^{2}={\mathrm{FWHM}}_{\mathrm{dp}}^{2}-{d}_{\mathrm{foil}}^{2}$$

The higher depth resolution for fluorescence lines of higher energy agrees with the indirect proportionality of the critical angle of total reflection (polycapillary optics) and the photon energy [42]. In praxis, the energy-dependent transmission efficiency of the optics at synchrotron setups is determined by measuring the photon flux before and after polycapillary optics [15, 25]. The reconstruction model by Lin et al. was evaluated quantifying a certified reference material (CRM) glass (NIST SRM 611). Much more homogenous distribution maps were achieved by applying the quantitative method. Statistical fluctuations were considered to be caused by varying physical properties like the density. In this work, the effectiveness of quantitative calculations was verified successfully [25].

The development of quantification routines of tabletop setups using an X-ray tube for excitation requires adapted calculations, due to the polychromatic character of the incident beam. One of the first quantification routines for stratified samples was described by Mantouvalou, calibrating the spectrometer with thin single-element foils, glass standards (NIST 1412, Breitländer BR D3), and an alloy brass standard (BAM M387). The calculation was based on a modified fundamental parameter equation [57] under the assumption that photoionization is mainly caused by the characteristic lines of the excitation spectrum. However, this is not applicable for all laboratory setups, since transmission of a polycapillary full lens behind the radiation source dampens the intensity of K fluorescence lines. Therefore, Förste et al. presented an adapted and generalized calibration procedure by implementing suitable software tools for fast deconvolution aiming for calibration and quantification routines [28]. The approach strived for a full quantitative reconstruction of the sample composition and structure by detecting the fluorescence intensity as a function of a three-dimensional position, considering the self-absorption of excitation and stimulated radiation. This was achieved by implementing the peak intensity as a function of concentration, overall density, energy of absorption, maximum excitation energy, energy dependent excitation intensity, photo production cross section, integral sensitivity, linear absorption coefficient, probing volume size, surface position, and thickness of the sample. Furthermore, the integral sensitivity, the probing volume size, and the spot sizes (decaying exponentially) were defined [58]. The transmission was described (in approximation) by a Gumbel distribution of both lenses, and combining transmission of excitation and fluorescence energy, finally, resulted in eleven free parameters, which were derived by calibrations. By measuring the depth profiles of known reference samples, the spot size and transmission of lenses could be characterized. Through these, 3D functions of integral sensitivity and probing volume size were assessed. For 3D quantification, a sample model containing sample geometry, density, and composition was used and the sample parameters were fitted to the best fitting of the experimental data. The calibration measurements were carried out on pure element reference samples (for transmission in excitation channels), razor blades and thin metal foils (for spot size in excitation channel), and thick glass multielement samples (for calibrating the probing volume). In order to validate the procedure, pulverized standard reference materials (SRM) and stratified polymer samples were measured [59]. The calibration was executed in an energy range from 3.3 keV (for K) to 14.9 keV (for Y), subsequently, plotting the probing volume size (characteristic exponential decay of the spot size directly visible) and integral sensitivity (following mainly the Gumbel distribution of the transmission) for excitation and detection energy. The quantification of element contents in the SRM and glasses showed deviations around 30%, which originated from the statistical uncertainty of measured trace elements, the overlap of fluorescence lines, and the SRM inhomogeneities. On the other hand, the quantification of the layered samples, with exactly known concentrations in each layer, achieved deviations below 10% from the certified values for concentration. Hereby, the influence of planarity of the layers, inhomogeneity of the element distributions, suitable layer thickness with respect to the lateral resolution of the CMXRF, and the relatively high age of the samples were listed as causes for the deviations [28].

Another quantification approach for CMXRF is possible by using Monte Carlo (MC) simulation, which also offers the opportunity to consider interfering effects like scattering effects or secondary fluorescence [60]. During MC simulation, the destiny of every single photon and its interactions are simulated. Several pathways of the photons are described, like the excitation of fluorescence radiation and inelastic and elastic scattering as well as further secondary interactions of the photons. The final summarization of all generated pathways of the photons provides a simulated XRF spectrum. Beforehand, the MC algorithm requires various parameters to be specified, like chemical composition, density, and sample size as well as experimental and fundamental parameters. Czyzycki et al. successfully developed a MC simulation code (monochromatic excitation) for the determination of layer thicknesses and element concentrations of various foils and multi-layer samples by deconvolution of the depth profiles [61, 62].

Stratified polymeric reference materials were studied during the past 15 years for the verification of CMXRF spectrometers and the applied quantification methods [14, 46, 59]. For example, Wrobel et al. successfully applied inhouse standards developed as polymer multilayer samples for validation of a proposed reconstruction procedure for stratified materials investigated with polychromatic radiation [54]. For exact evaluations of such quantification routines, inhouse reference materials of very high homogeneity should be applied. A recent and reproducible preparation of such polymeric stratified standard materials was presented by Rogoll et al. using filler materials which were solvable in the polymeric matrix. Hereby, the optimization of the layer preparation was necessary to achieve a very high homogeneity of the element distribution in the polymer as well as constant layer thicknesses. The successful implementation for CMXRF analysis was demonstrated and evaluated by several multilayer samples containing iron filler materials [27].

Light organic matrices are particularly suited for CMXRF measurements, because in these materials, absorption effects have less influence on the measurements. Due to this fact, the majority of applications up to now was shown for samples with light matrices, for example, in the qualitative examination of art-historical objects, such as parchment sections, painting layers, or polymers in materials analysis [26, 46, 55]. In addition, CMXRF is suitable for the investigation of plants, for example, to follow up the nutrient uptake of parasitic plants via host connections [63] or for the determination of inclusions in diamonds and silica-based minerals in the field of geosciences [28, 42].

## Applications of CMXRF spectroscopy

Due to the non-destructive character of the CMXRF, the relatively small sampling volume and the rather good sensitivity, even when performing a 3D experiment, this technique has high potential for applications in materials and geosciences, biology, and archaeometry, mostly for matrices with low or moderate average *Z* numbers, such as

- The analysis of samples from the interdisciplinary field of materials science, like car paints, industrial plastic materials, electronics, and catalysator materials. CMXRF can be used to determine the elemental composition of the samples, providing insight into the sample structure, and consequently can help to connect sample structure with their properties and behavior.
- The analysis of historic artifacts and samples, including paintings, writings, ceramics, glasses, and mortal remains. The 3D technique can be used to determine the elemental composition of these artifacts, providing valuable information about their age, origin, and history of making.
- The analysis of biological samples, including tissues and cells. The determination of the elemental composition of these samples provides insights into their structure, function, and even potential health impacts.
- The analysis of geological samples, especially liquid and solid mineral inclusions. Here, the elemental composition of these samples can provide valuable information about the processes of mineral genesis and allows acquiring information about the composition of deep-lying rock strata.

### Materials science

**Fig. 3:**
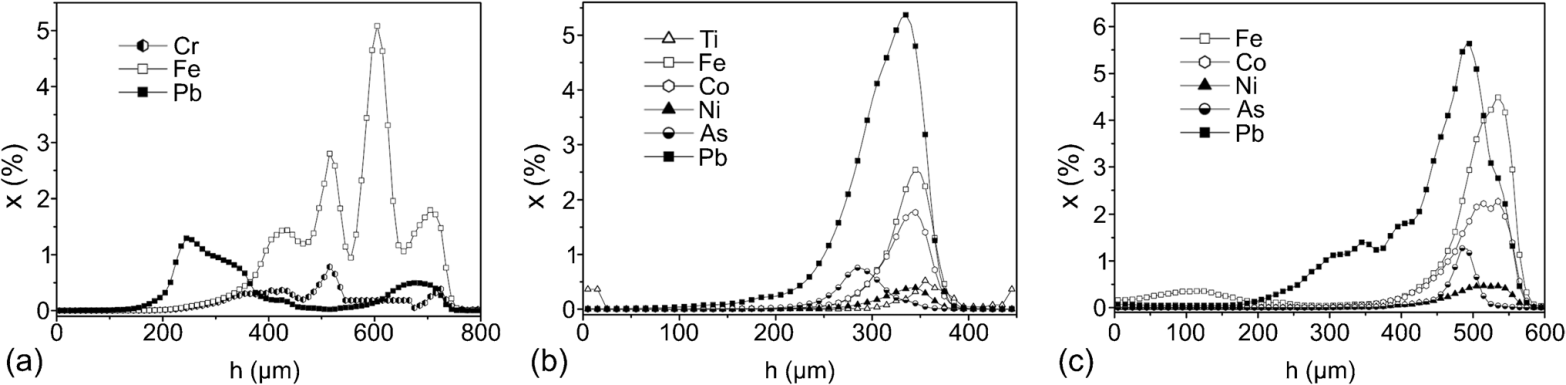
Depth scans (*y*-axis: weight percentage) of three different car paints. (**a**) Five-layer paint, (**b**) two-layer structure imbedded in lead-white matrix, and (**c**) four-layer paint. Adapted from reference [24] with permission from Elsevier

The application of CMXRF for material analysis started as soon as the development of this analysis technique. Šmit et al. measured in 2004 different types of car paints with a confocal setup at the HASYLAB beamline L. Between the excitation X-ray beam of 21 ± 1 keV and a Si(Li) X-ray detector, two identical X-ray lenses were arranged in confocal geometry creating an approximate interaction volume of 30 µm. The confocal analysis of the car paints, performed in 10 µm scan steps in *z*-direction showed sequences of a multitude of layers. Figure 3 shows three quantified depth scans of those car paint samples for different paints and elements, respectively. The applied quantification procedure took the absorption of X-rays and secondary fluorescence into account and yielded correct concentration ratios. The overall thickness of the samples was up to 500 µm, with individual layers exceeding 30 µm thickness [24].

Besides a chemical microchip, Nakano et al. also investigated an industrial plastic sample in order to identify hazardous contaminants produced within the process of recycling, since limitations of those contaminants were laid down in international agreements in order to prevent adverse health and environmental effects. The applied tabletop spectrometer consisted of an X-ray tube with a Mo target, a polycapillary full lens in the excitation channel, a half lens in the detection channel, and a SDD for X-ray detection. Depth profiles indicated that the plastic sample consisted of a thin green paint layer which was well distinguished from the base plastic. The base plastic mainly contained Ca and Br which have been added for flame retardant and stabilizer reasons, whereas within the green paint layer, Pb, Cr, Ti, and Cu were detected. The authors assumed that yellow (PbCr_2_O_4_), green/blue (Cu-phthalocyanine), and white (TiO_2_) pigments were incorporated into the green paint layer [46].

In a recent work, Nakano et al. presented a technical adaption of the classic tabletop confocal XRF spectrometer with the capability for spot analysis. Instead of polycapillary lenses, Soller slits were implemented for aligning the incident X-rays and generated fluorescence radiation, creating a so-called confocal line XRF system. This spectrometer can be used for analyzing stratified samples. In this explicit study, a leather sample (Cordovan), provided by the Department of Research of the Hyogo Prefectural Police, was investigated. The dyed side of the sample was rich in Zn and the bulk material was rich in Fe. With the confocal line XRF, a 33-fold intensity increase was achieved in comparison to the original confocal point XRF. Thereby, the lateral resolution of the confocal line setup was lower but could be adapted by using thinner slits [56].

Another recent work dealt with the investigation of silica-based anisometric supraparticles with catalytic active patches for self-propulsion on the basis of Pt-covered magnetite (Fe_3_O_4_) nanoparticles. Oguztürk et al. measured carbon tape–fixated supraparticles by the CMXRF laboratory setup at the TU Berlin. The setup consisted of a Mo microfocus tube, two polycapillary lenses, and an SDD. Due to the low density of the supraparticles, the localization of Fe and Pt was performed without the need for sectioning. Multiple element distribution images at varying *z* coordinates were measured (Fe, Pt, and scatter radiation), giving an insight into the composition of the particles. Without a magnet, the catalyst was distributed evenly within the supraparticles. The results showed that the Pt-covered magnetite nanoparticles were embedded quite deep in the supraparticles and exhibited a well-defined structure. With a magnet, the patch was localized in a small volume determined by the position of the magnet. After a joined application of a magnet and additional components with a high network characteristic, Fe/Pt were distributed much more heterogeneously over a large volume, because of the impeded nanoparticle movement. It was stated that the well-defining of the resulting patchy will change the propulsion properties of the particles. CMXRF enabled the 3D investigation of the internal structure and distribution of the nanoparticles with a resolution of about 30 µm [64].

Last but not least, Bauer et al. investigated dentin surrounding fillings. The background of this work was the quantification and better understanding of the interactions between dental restorations and tooth tissue over time by analyzing a root canal–treated tooth, which underwent multiple dental treatments, and several freshly treated teeth. Measurements were carried out with µCT, SEM-EDX, and CMXRF. Two different CMXRF setups were used: a modified Bruker M4 Tornado with an Rh anode and a flexible spectrometer with a Mo anode. The depth resolution varied from 60 µm for Ca K fluorescence to about 10 µm for high-energy fluorescence lines. The total analysis volume of the re-treated tooth was 720 × 1710 × 240 µm^3^, which covered an area between the root canal filling and the root in order to analyze the Zn distribution in depth, since it was observable within the internal root canal as well as deeper within the root walls in faint traces. The CMXRF measurements proved that the Zn distribution was not an artifact generated by sample slicing, since Zn fluorescence detection exceeded 1500 µm. Furthermore, line profiles of the depth measurements exhibited high Zn values at depths up to 300 µm from the interface between the dentin and the filling. For a 100 µm span, the Zn signal was low, due to a zone rich in Ba and Bi, and at around 400 µm, increasing Zn values were observed, which decreased gradually with increasing distance from the canal. It was stated that some of the Zn from the filling diffused through the dentin into the tooth tissue, whereby this zinc originated from an earlier treatment, possibly from a Zn(OH)_2_ dressing, which is recommended prior to root canal treatment. Hereby, CMXRF contributed important insights in the depth element distribution within the tooth and the filling materials (efficient for > 3 keV fluorescence lines) without being effected by surface artifacts due to the preparation [9].

### Archaeometry

Archaeometry is one of the very prominent application fields of CMXRF and was also approved from the beginning for all sorts of samples, like paintings, manuscripts, ceramics, glasses, and mortal remains. CMXRF enables non-destructive information about the elemental composition and though allows interpretation about the samples’ origin, preparation methods of the artifacts, forensic evaluations, and many more. In particular, the non-destructive character is very important for the analysis of samples of high cultural and historical importance, which should stay untouched.

Guilherme et al. presented the first analytical results on Portuguese ceramic pieces in 2011, which were produced in Coimbra and Lisbon between the sixteenth and eighteenth centuries and were assigned beforehand by art historians on stylistic basis. In this work, XRF and SEM-EDX results exhibited different manufacturing processes for the two production centers by analyzing the glaze and pigment thicknesses and investigating interfaces of color-glaze and glaze-ceramic, since the dissemination of abundant elements is dependent on compositions, pigments, and temperature. CMXRF measurements were carried out with a tabletop setup consisting of a 30 W Mo microfocus tube, an SDD, and two polycapillary lenses (full and half lens) with a 90° angle between the optics and 45° angle between sample normal and detector axis [51]. Due to high absorption effects by the high amount of lead in the samples, the depth sensitivity was quite restricted, so the probing volume was kept close to the surface of the sample. The confocal geometry applied and the restriction of the analyzed volume to the region close to the surface resulted in reduced scattered radiation. The evaluation of such surface scans allowed insights into compositional and microtextural data of both groups of the samples, revealing the different properties of the glazes. In some cases, the interfaces between frit (pre-melting of pigments with fluxes, like Pb, Na, Sn) and base glaze were not distinguishable, so it was assumed that the frit and glaze had higher fusibility and/or the firing temperature was higher. This allowed trace back and to distinguish both groups of ceramics in favor of their production site [65].

**Fig. 4:**
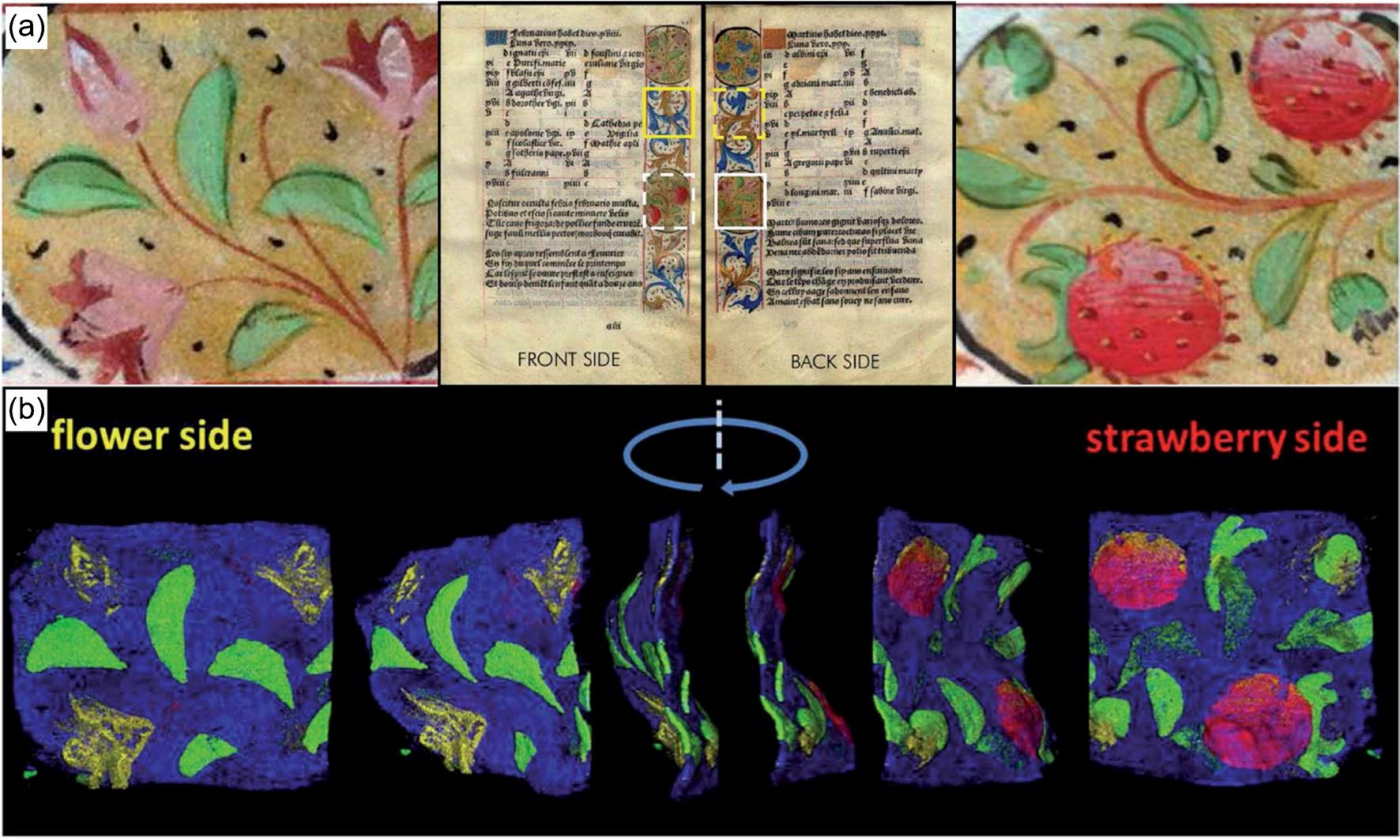
(**a**) Parchment sheet with analysis area “strawberry” (dashed white outline) on front side and “flower” (white outline) on the back side. (**b**) Different views of the composite 3D distribution with Hg Lα (red), Cu Kα (green), Au Lα (blue), and Pb Lα (yellow) and stretched depth axis by factor 10. Adapted from reference [26] with permission from the Royal Society of Chemistry

The full separation of front and backside decorations of an illuminated parchment manuscript was achieved by CMXRF after the 3D examination of sections of the manuscript depicted in Fig. 4(a) with white lines and the zoomed representations of strawberries on the front and flowers on the backside. For the measurements, a Bruker M4 Tornado (MXRF) was used, which was modified with a second polycapillary in front of the detector with an 80° angle between both optics. The spectrometers’ probing volume decreased in *z* direction from 60 µm for Sn Lα to 20 µm for Zr Kα with the highest sensitivity for fluorescence energies between 4 and 9 keV. By applying three-dimensional measurements, imaging of pigment layers on the front and backside was achieved. In total, 87 slices with a 15-µm step size were measured and evaluated. Data interpretation was complicated by the fact that the parchment was not perfectly flat. By careful data processing and subsequent evaluation, a full separation of both pigment layers was achieved. By arranging the individual slices, the 3D reconstruction in Fig. 4(b) of the selected parchment section was achieved for Hg Lα in red, Cu Kα in green, Au Lα in blue, and Pb Lα in yellow. By this non-destructive analysis and the presented 3D evaluation method, it became possible to reflect the decoration without ambiguity. Therefore, this application shows the importance of CMXRF analysis for cases in which the sample is so fragile, so the sample backside could only be measured by non-destructive and depth-sensitive analysis techniques [26].

Reiche et al. investigated paint layers of the Renaissance paintings series, *Famous Men*, from the Louvre collection. Hereby, the presence of multiple thin layers was a great challenge. The applied tabletop CMXRF spectrometer LouX^3D^ allowed non-destructive and depth-resolved analysis with a setup consisting of an Rh tube, a polycapillary full lens in the excitation channel, and a polycapillary conical collimator in front of an SDD for detection. The depth resolution of the confocal probing volume ranged from 34 µm (Zr Kα) to 58 µm (Ti Kα). The energy-dependent probing volume size and the energy-dependent absorption of fluorescence radiation were considered for qualitative interpretation of the measured depth profiles. CMXRF depth profiling revealed overpaints for the portraits of Plato and Aristotle. For the Plato painting, an Fe- and Pb-containing red layer was identified, which covered a Cu-containing green layer, depicting Plato’s dress. Moreover, it could be stated that Aristotle wore a red stole (Hg-based layer, which was attributed to vermilion), which was overpainted with a purple color (Pb-based layer containing Ca, Zn, and Fe). The future archaeometry-based applications of this CMXRF setup were stated to be the analysis of additional paintings to gain information about historical art objects, identify repaints, and optimize restorations and the reconstruction of the original painting [66].

Another paint-layer investigation was performed by Prokeš et al. based on the analysis of Bohemian panel paintings. The applied laboratory setup consisted of a Mo target X-ray tube with an attached polycapillary lens, a polycapillary collimating optic as secondary lens, and an SDD for detection. The setup shows typical depth resolutions for a tabletop setup within a range of 27 ± 2 µm for Pb Lα and 46 ± 2 µm for Ti Kα. With this setup, the authors were able to analyze the composition and structure of pigments containing layers, which were rather thin with a thickness of only a few tens of microns. Hereby, the used pigments within the paint layers were determined, like lead white, vermilion, ocher, and calcium carbonate, thus contributing to the analysis of historical, medieval paintings. Comparing the depth results obtained before with those from the CMXRF analysis of Lanna’s Assumpta painting, the results confirmed a good correspondence with the technique characteristics for the Bohemian panel painting. Therefore, it was stated that both paintings originated from the same workshop [55].

### Biology

Besides archaeometry, applications for CMXRF are also found in the field of biology, due to often quite light matrices of the samples, which guarantee relatively high analysis depth combined with a high fluorescence radiation yield for elements with higher *Z* numbers. Since absorption effects are sometimes nearly neglectable, far easier qualitative and quantitative approaches are possible for data evaluation.

Van Malderen et al. investigated the three-dimensional elemental distribution of elements like Cu, Ni, and Zn within the freshwater crustacean *Ceriodaphnia dubia* (*C. dubia*) by applying different analysis techniques like LA-ICP-MS, µCT, and CMXRF. In this work, the focus was set on uptake efficiency, chronic toxicity, and tissue-specific distributions of trace elements. The CMXRF measurements were performed at the former beamline L of the DORISIII synchrotron at the DESY in Hamburg, Germany. For excitation, the monochromatic high-intensity radiation of 15 keV was used in combination with a polycapillary optic for focusing. The confocal geometry was achieved by implementing a second polycapillary optic in front of the detector. The setup allowed the analysis of chemically fixed and air-dried *C. dubia* without the need of sectioning. The elemental isosurface thresholds of the CMXRF scans were adapted to a µCT rendering of the sample. Manganese distributions were correlated with the regions of hepatopancreas, gut, and eggs; iron and zinc could be identified within the osmoregulatory tissue. In combination with the 3D results of µCT and LA-ICP-MS, CMXRF contributed first information about the element distributions to specific tissues within *C. dubia* [6].

Van der Ent et al. examined samples of the fern *Pteris vittata*. This fern specimen exhibits extreme arsenic hyperaccumulation characteristics. Thereby, this study provided insights in the chemical speciation and arsenic distribution within cell types measured by XANES and XRF analysis. CMXRF measurements were carried out at the X-ray fluorescence microscopy (XFM) beamline of the Australian Synchrotron delivering a brilliant X-ray beam (15.8 keV) with a focus down to 1000 nm by using a Si(111) monochromator and a pair of KB mirrors. The confocal arrangement was achieved by implementing a custom-made polycapillary optic mounted to an SDD resulting in a depth resolution of about 10 µm. The frozen-hydrated fern samples were measured under a cryo-stream whereas the freeze-dried samples were analyzed at room temperature. Confocal cross-section (*x*–*z*) measurements of the central part of the pinnule showed that arsenic was strongly accumulated in the endodermis and pericycle surrounding the phloem. Furthermore, it was observed that As accumulation took place in the abaxial epidermal cells of the midrib, in the indusium, and in the sporangia, particularly the annulus [13].

The publication of Förste et al. [63] dealt with the analysis of infection sites by rootless parasitic plants, which bond to a host for nutrient supplies. Besides other measurements, CMXRF analysis delivered results about element concentrations at the host/parasite interface. The setup consisted of a commercial MXRF spectrometer (Bruker M4 Tornado) which was equipped with an Rh X-ray tube, a polycapillary lens, and an SDD. By the perpendicular arrangement of a second polycapillary optic in front of another SDD, the confocal geometry was achieved with a resolution of 30 µm for Fe Kα. This exact setup was also used before by Mantouvalou et al. for the analysis of two genotypes of pearl millet seeds, as a first step toward 3D element quantification in the grains of crop plants for the comparison of mineral contents in different grain genotypes [67]. For the analysis of the host/parasite interface, a high-resolution confocal scan of the endophytic haustorium and surrounding tissue was executed within a total analysis volume of 1550 × 450 × 1000 µm^3^. The 3D scan showed a depletion of Ca and Mn and an enrichment of K in the haustorium. Furthermore, a rather thin layer of Mn engulfing the haustorium was observed. Hereby, CMXRF analysis allowed the 3D depiction of heterogeneously distributed elements in the different regions of the investigated infection site [63].

**Fig. 5:**
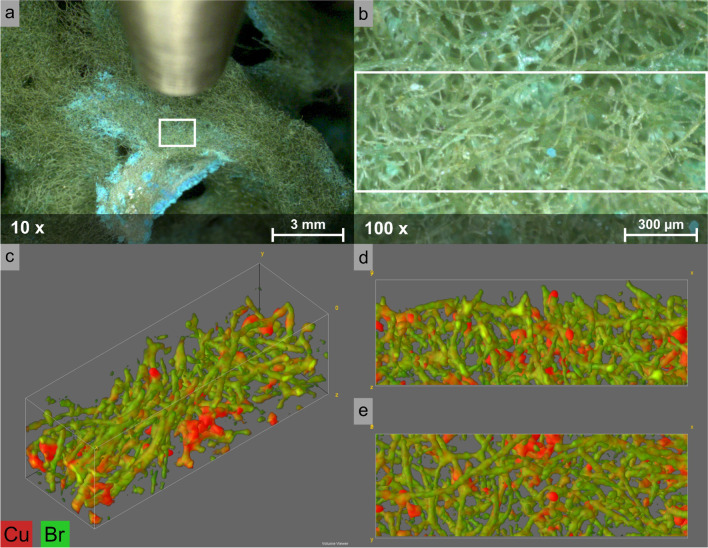
A spongin-based porous microfibrous scaffold of *Hippospongia communis* bath sponge, after being placed in a model ammoniacal CuCl_2_ solution, was covered with a layer of green crystalline material (10× magnification (**a**), 100× magnification (**b**)), which has been identified in this study as atacamite [68]. (**c**) 3D distribution, (**d**) the side, and (**e**) top view of copper and bromine distribution in the spongin–atacamite composite, measured in a part of the sample with dimensions of 1.5 × 0.5 × 0.5 mm^3^

Sponges are a very versatile group of specimens with manifold properties and behaviors. Due to their porous and scaffold-like structure, three-dimensional analysis techniques can deliver important information about the spongin’s three-dimensional elemental composition. In respect to CMXRF analysis, two different samples were examined, a spongin-based scaffold of *Hippospongia communis* by Tsurkan et al. and the marine demosponge Giant *Ianthella basta* by Kertmen et al. [68, 69]. The applied spectrometer for both publications was a modified Bruker M4 Tornado with a microfocus Rh X-ray tube and a 60 mm^2^ SDD. The confocal arrangement resulted from a polycapillary full lens in the excitation and a polycapillary half lens in the detection channel with a 50° angle to the sample normal. The spongin-based scaffold of *H. communis* was treated with a model alkaline, toxic Cu waste solution. The aim of this work from the field of extreme biomimetics was to develop high-performance 3D structural composites with enhanced multifunctional capabilities on the basis of already-existing macroporous bioarchitectures generated by natural polymers. In the experiments, the formation of a green crystalline material (atacamite) covering the spongin scaffold was observed (Fig. 5). CMXRF measurements allowed a three-dimensional depiction of the Cu and Br distribution within the spongin–atacamite composite for a total analysis volume of 1500 × 500 × 500 µm^3^, visualizing the spongin structure (Br) and the formed atacamite (Cu) [68]. On the other hand, for identification of mineral phases within chitinous skeletal structures of Giant *I. basta*, the marine demosponge was treated with distilled water, HCl, and NaOH solutions, respectively. CMXRF analysis was used for the 3D visualization of the structurally inhomogeneous samples as well as the acquisition of information about the distribution of bromine, which originates from halogenated compounds within the spongin, and calcium present as nanocrystalline calcite. Examinations showed that the calcitic nanocrystals of the biomineral phase were dissolved by acidic treatment with HCl [69].

As the last example in this chapter, investigations of Zoeger et al. on lead accumulation in tidemark of articular cartilage are discussed [70]. Here, different spatially resolved analytical techniques were applied, studying the microscopic distribution of Pb in the chondral and subchondral region from normal humans in the context of chronic diseases associated with Pb accumulation in the skeleton. CMXRF measurements were carried out at HASYLAB, beamline L in Hamburg, Germany, with polycapillary half lenses in the excitation and detection channel. This setup was applied for area scans (400 × 400 µm^2^) as well as a 3D volume scan (200 × 200 × 160 µm^3^) on a patella slice. The executed analysis in confocal geometry exhibited Pb accumulation within the transition zone between the non-calcified and calcified articular cartilage (tidemark). The tidemark represents the metabolic calcification front for bone formation. It is assumed that, due to the possible displacement of Ca^2+^ with lead by cation exchange, the material property of the bone could be altered. In this study, the first 3D representation of the Pb distribution in the tidemark was presented, which provided an important first step in the investigation of Pb accumulation in articular cartilage [70].

### Geosciences

Most of the applications in the field of geosciences were dealing with the three-dimensional analysis of mineral inclusions and particles. In one of the first papers in this field, Janssens et al*.* analyzed in 2004 the distribution of iron-oxide grains in a polished granite sample (thickness of 100 µm) containing Fe and Sr [43]. Vincze et al. successfully analyzed 3D trace element distributions in solid inclusions (Sr, Zr, Th) in diamond as well as in fluid inclusions (Fe, Cu, Zn, Ga) in quartz [42].

Bauters et al. applied a confocal MXRF and XAFS spectroscopy setup at the European Synchrotron Radiation Facility Collaborative Research Group Dutch-Belgian Beamline (ESRF CRG BM26A) for the analysis of deep-earth diamond inclusions. The synchrotron beam was focused by a polycapillary optic to a size of 8 × 8 µm. The confocal volume was created by a second polycapillary optic in front of an energy-dispersive X-ray detector. The non-destructive analysis of the diamond inclusions contributed information about the encapsulated material from the earth’s mantle transition zone in respect to the diamond formation. After a 2D XAFS mapping of the inclusion and the analysis of eight Fe standards and minerals, the inclusion was determined to be 91% olivine with 9% Fe_2_O_3_. CMXRF measurements in a 170 × 170 × 70 µm^3^ volume were successfully performed on a different inclusion, confirming the three-dimensional sensitivity of the confocal setup presented in this work [15].

Recently, Lin et al. demonstrated the capabilities of CMXRF measurements to investigate fluid inclusions, consisting of a liquid and a vapor phase, within a natural beryl crystal by a synchrotron setup at the hard X-ray micro-focusing beamline (BL15U1) of the Shanghai Synchrotron Radiation Facility (SSRF) using a Si(111) monochromator (energy of 17.0 keV) and a KB mirror system. The confocal arrangement was achieved by implementing a polycapillary optic in front of a 50 mm^2^ SDD in a 90° angle to the incident radiation, resulting in depth resolutions of 10.2–26.5 µm in the energy range of 5.0–13.0 keV. The beryl crystal was prepared as a 300-µm-thick double-polished thin section, containing several fluid inclusions with sizes > 20 µm. The inclusion of interest was located about 60 μm beneath the samples’ surface. Bromine fluorescence radiation was detected within this fluid inclusion, and the Br distribution perfectly outlined the shape of the aqueous phase. Cu, Zn, and Ga were homogenously distributed in the beryl crystal (Zn and Ga) or concentrated at the rim of the crystal sample (Cu). CMXRF analysis and data quantification allowed the visualization of the distribution of different phases and the spatially resolved data interpretation [25].

Another mineralogical application by Förste et al. [31] represents an approach to quantifying a mineral inclusion based on quantification routines for full three-dimensional elemental distributions by calibrating laboratory spectrometer parameters. In this work, the established quantification technique was validated by measurements of SRMs and polymeric multilayer systems with a CMXRF setup (BLiX setup) and was approved by measurements at a similar setup (Freiberg setup). Both setups, based on a modified commercial spectrometer (M4 Tornado), provided 3D resolved element analysis by the implementation of an additional polycapillary lens. The BLiX setup was equipped with a 50 W and the Freiberg setup with a 30 W Rh-microfocus tube. Additionally, a vacuum of 20 mbar was applied at the Freiberg setup, increasing the sensitivity for light elements (with respect to the Si K fluorescence line). The 3D calibration of the spectrometers was executed with depth profiling measurements of multi-element glass samples. The investigated sample was a smoky quartz with a ferrous inclusion. For the reconstruction procedure, a multilayer system with quartz in the first and third and goethite in the second layer was assumed. Furthermore, a scattering peak at 9.9 keV was used for determination of the quartz surface. Hereby, the 3D reconstruction supplied additional information on the position and size of the goethite inclusion by correction of absorption effects and the different probing volume sizes (fluorescence lines of Si K and Fe K). The successful three-dimensional visualization (although quite different matrices and densities were present) approved the future applicability of laboratory CMXRF setups for the examinations of geoscientific samples [28].

## Challenges of CMXRF spectroscopy

Despite the promising developments during the past years, there are still several challenges associated with CMXRF spectroscopy which have to be addressed to mature this technique for routine applications.

Some of these challenges are not unique for CMXRF but rather typical for methods which aim to obtain information from micro- or nanoscalic sample regions. One of those is the importance of sample preparation for inhomogeneous materials with regard to selecting representative sample regions and artifacts from certain preparation steps. This is especially crucial for samples with complex geometries or multiple layers where the alignment in the measuring chamber is also important for good results. Another one is the crux between necessary and available spatial resolution. Since in the case of CMXRF the resolution depends on several parameters but particularly on the atomic number of the investigated element, one has to determine this parameter for each element separately. In addition, mostly due to geometrical requirements, tabletop instruments using X-ray tubes as excitation source are actually restricted to the application of capillary optics and consequently provide rather worse resolution in comparison to synchrotron constructions. The energy-dispersive measurement of the fluorescence signal from the sample leads often to spectral interferences of neighboring fluorescence lines, especially when elements with medium and high *Z* numbers have to be measured in the same sample. Secondary fluorescence effect generated by matrix components can affect the accuracy and reliability of CMXRF measurements.

One of the challenges which are generated by the geometry of the setup and the devices placed in the optical path is the decreasing sensitivity with increasing depth. Since this is also strongly influenced by the atomic number of the elements involved, one can end up with inconsistent or incomplete information about the sample composition at higher depth. In cases of large differences in *Z* between the elements investigated, reconstruction of the real composition will be only successful for the region close to the sample surface. For greater sample depth, information about elements with lower atomic numbers get lost, because X-rays of those elements are not able to reach the detector thus resulting in incomplete reconstruction of composition. The increasing X-ray absorption of elements with higher atomic numbers also generates the effect, that the average information depth depends on the average *Z* number of the sample material, and for materials with higher amounts of heavy elements, the information depth is often found around few tens of microns. This is also one of the reasons why CMXRF applications presently are mainly restricted to samples with light matrices. Last but not least, for very inhomogeneous materials, an accurate characterization and quantification of the chemical composition can be challenging, because the quantification models actually available do not take strong differences in density on the microscale into consideration, though this parameter has an influence on the X-ray absorption process and consequently on signal intensities of lower sample zones. Connected with this is the need for well-defined inhomogeneous reference materials which could be used for the calibration of the instruments, testing the mathematical models, and finally, evaluating the data from sample measurements. Unfortunately, here exists a deficit which could only be overcome by producing such samples by themselves.

To overcome existing shortcomings of the method, several approaches are possible. Generally, higher intensities in the excitation process will be beneficial to obtain information from greater depth. This can be achieved using a specialized X-ray tube with higher accelerating voltages and/or higher power (50 W) or by other sources, e.g., liquid metal sources, which are presently tested, but are rather costly and require specialized expertise to operate. Interferences due to spectral overlap could be reduced by applying spectrometers with higher resolution. This is more easily done at synchrotron facilities or homemade tabletop instruments, but needs cooperation and persuasion with companies building MXRF instruments for incorporation into existing commercial systems. To improve the evaluation process for measured data, specialized software routines are necessary which could be applied routinely at commercial instruments. Right now, models are under development which will improve data treatment at instruments working with polychromatic excitation sources.

## Outlook for CMXRF spectroscopy

CMXRF spectroscopy is already a powerful non-destructive analytical technique with many applications in a variety of fields where spatial resolution in 10–50 µm dimension provides insight into the composition of inhomogeneous materials. Maturing into a routine technique will depend on the development of suited sample preparation techniques and the implementation of new instrumentation and software into homemade tabletop or commercial instruments.

Efforts to gain in sensitivity have been made by different groups during the past 3 years. One example for this is the recently developed confocal line spectrometer, where the adjustments of a confocal XRF spectrometer lead to higher net signal intensities (sensitivity) due to the created line profile of the probing volume instead of an ellipsoidal or a confocal point setup. The group developing this type of spectrometer is also working on quantitative procedures for this setup [56]. A liquid metal jet source (LMJS) was implemented as a new type of X-ray source. By adapting the parameters of the focusing lenses to the LMJ source, a significant intensity enhancement (gain in sensitivity by factor ~ 60) for transition metals was achieved [58]. This resulted in reduced measuring times and an easier quantification due to assumed monochromatic excitation by the LMJS for 3D element distribution images [71]. Here, the future developments aim for X-ray sources with a higher radiation yield and overall source brightness, an optimized adaption of the implemented (polycapillary) optics for higher lateral resolution [48], and an enhanced sensitivity of state-of-the-art detectors (predominantly SDD). Other steps consist of the application of a vacuum or He atmosphere for a better sensitivity of light elements or the implementation of more automated routines, e.g., optimal optic alignment and spectrometer calibration [28]. Another advance is the combination of CMXRF with additional analytical techniques to enhance or correlate the analytical information about the sample, e.g., by a combination with computer tomography or Raman spectroscopy [9, 72]. Also promising is the development of 2D/3D full-field XRF (FF-XRF) techniques by applying a new type of CCD-based 2D detector. Hereby, the lateral resolution can be influenced by the used optics and the analysis time is reduced by half in comparison with CMXRF, due to the simultaneous spectra acquisition for each image pixel [73, 74]. Furthermore, in the context of FF-XRF, the application of coded apertures was studied and resulted in higher count rates and potentially higher spatial resolution [75]. Possibly, these or comparable methods could be applied to CMXRF analysis in the near future.

Also, further developments in the sector of spectrometer calibration and data quantification are to be expected. Förste et al. contributed to the acceptance of laboratory CMXRF and its quantitative routines only recently [28] with a quantification model for polychromatic sources, for which relatively flat and laterally homogenous samples (like layered samples) were assumed. The boundaries were set for samples with edges, high-density differences, secondary fluorescence, and other higher-order effects. The next steps the group is working at are the implementation of information about scatter effects and of different geometrical structures, like edges or inclusions, in quantification routines [28]. Another approach to improve FP-based quantification procedures addresses the implementation of secondary effects such as fluorescence enhancement and the fundamental problem of not collected emission due to the absorption at deeper sample regions and/or the low fluorescence energies of light elements [25]. Of high interest for all fields of applications is also the development of suitable reference materials, e.g., stratified materials of polymeric matrix, which could be used for the calibration of CMXRF setups and other depth-sensitive analysis techniques and for quantification of real samples. These materials should be characterized by a high vacuum and long-term stability, the ability to select elements of choice to implement into the materials, an excellent homogeneity on the micro- and nanoscale, an available concentration range from weight-% down to ppm and possibly ppb, the opportunity to realize variable layer thicknesses, and a good replicability of the manufacturing procedure. These idealized demands are not easily met, but could already be partly accomplished by the model systems based on polyacrylates introduced for a wide selection of elements [27]. With these systems, future development of geometrically complex reference materials, e.g., samples with edges, inclusions, or concentration gradients, can be expected.

CMXRF spectroscopy holds the potential for continued development and expansion of its applications. This will be noticeably supported by the development of more advanced spectrometers, which can provide higher sensitivity and better energy resolution for the analysis, and by setups that may improve the spatial resolution and depth sensitivity of the technique, allowing for even more detailed mapping of elemental composition within a sample. With these developments, samples with higher average *Z* numbers will be included in the set of applications, thus widening the applicability of CMXRF in the fields of materials science, biology, geoscience, and archaeometry or expanding the use of this technique to new fields.

Synchrotron-based CMXRF or SIMS will still be competing methods in the future, mainly due to shorter analysis time and better spatial resolution. Nevertheless, in the first case, tabletop equipment provides easier access to the measurement; in the latter case, the spectra provided by CMXRF are much less complex and the sample will not be destroyed. There exists no method that can solve any analytical problem, not even CMXRF. Nevertheless, in the fields of applications on which we have focused here, this technique will become a very useful tool for answering questions connected with the three-dimensional sample composition, thus helping to better investigate complex and inhomogeneous materials.
